# Feasibility and acceptability of COVID-19 self-testing in the Philippines

**DOI:** 10.5588/pha.23.0045

**Published:** 2023-12-07

**Authors:** E. U. Mallari, S. Keller, J. F. Febre, J. Timbol, R. Powers, R. R. Peregrino, A. Slyzkii, C. Mulder, M. V. Magno, I. Spruijt

**Affiliations:** 1 KNCV Tuberculosis Foundation, Manilla, The Philippines; 2 KNCV Tuberculosis Foundation, The Hague, The Netherlands; 3 Aurum Institute, Accra, Ghana; 4 Amsterdam Institute for Global Health and Development, Amsterdam University Medical Centres, Amsterdam, The Netherlands

**Keywords:** The Philippines, Ag-RDT, acceptability testing, antigen-detecting rapid diagnostic tests

## Abstract

Self-testing for COVID-19 using antigen-detecting rapid diagnostic tests (Ag-RDTs) shows high promise in the Philippines. Self-testing has the potential to provide broader access to testing, empowering individuals by bringing healthcare services closer to them. We conducted 15 semi-structured interviews with health officers and decision-makers in the Philippines. These interviews explored the experiences and perspectives on the acceptability and feasibility of self-test use and implementation. We found that self-testing is easy-to-use, provides rapid results and can facilitate early detection. However, ­regulatory policies, linkages to care and effective health ­education plans must be in place for successful implementation.

In 2022, the Philippine Food Drug Administration (FDA) granted approval, and the Department of Health (DOH) recommended the use of self-testing for COVID-19 as a strategy to facilitate more rapid case detection and healthcare facility management.^1^–^3^ However, the Philippines continues to have a limited COVID-19 diagnostic and testing capacity, and case reporting is underperforming.^4^ Despite allowing the use of self-administered antigen-detecting rapid diagnostic tests (Ag-RDTs) in the Philippines, there is no study documenting its use, a vital necessity in creating policies for its implementation.

Information on implementation strategies was needed to accommodate roll-out. Hence, we assessed the perceived feasibility and acceptability of Ag-RDT self-testing among stakeholders in the Philippines. We defined feasibility as the extent to which COVID-19 self-testing could be successfully implemented and evaluated through perceived barriers and facilitators for implementation. We defined acceptability as the perception of self-testing being useful, agreeable or satisfactory in the local context.

Between November 2022 and January 2023, we recruited 14 stakeholders (6 males, 8 females, aged 28–66 years) using convenient sampling for semi-structured interviews (Table 1). Interviewees included five health officers (2 at the district/provincial level and 3 in the local administrative district, a *barangay*), 4 health system decision-makers at the municipal level, 3 decision-makers at the regional level, 1 national-level decision-maker and 1 member of a regulatory board. We obtained written informed consent from all participants prior to interviewing. Interviews were conducted in Filipino, audio-recorded, and lasted between 20 and 60 min. After verbatim transcription of the interviews, we translated transcripts into English. We used a thematic approach and NVivo 12 (Lumivero, Denver, CO, USA) for analysis. Identified themes and supporting quotes are presented in Table 2.

**TABLE 1:** Interview topic guide by theme

Theme	Example questions
Knowledge and perceptions of COVID-19 in general	Could you please explain what COVID-19 is? Could you explain who is most vulnerable to COVID-19? Could you explain what measures should be taken when one has COVID-19 symptoms?
Knowledge and perceptions of COVID-19 diagnosis	Could you explain how COVID-19 can be diagnosed? Could you explain where people in your area can go for a COVID-19 diagnosis?
Knowledge and perceptions of COVID-19 self-testing	Have you heard about COVID-19 self-testing before? Can you explain what COVID-19 self-testing is? What are the reasons for you to do a COVID-19 self-test? What would be the reasons for you not to do a COVID-19 self-test?
COVID-19 self-test result	Can you explain how you interpreted the result of the COVID-19 self-test? Did you trust the test result? Why/Why not?
Linkage to care following a positive self-test	Could you explain what kind of linkage to care is currently available in your country? Could you explain what policies still need to be developed to ensure this linkage to care in your country? Could you explain which factors could hamper the translation of such policy into practice in your country?
Current COVID-19 testing policies	Could you explain what current policies are in place in your country regarding COVID-19 testing? Could you explain which factors hamper the successful translation of that policy into practice? Could you explain which factors hamper the successful translation of that policy into practice?
Acceptability* of COVID-19 self-testing	What role do you think COVID-19 self-testing could have in the fight against COVID-19 in your country? What do you think are the advantages of COVID-19 self-testing? What do you think are the disadvantages of COVID-19 self-testing?
Feasibility^†^ and implementation of COVID-19 self-testing	What policies need to be developed for the self-testing to be rolled out in your country? What barriers do you see and expect in the implementation of COVID-19 self-testing among symptomatic presumptive COVID-19 patients? What factors would facilitate the implementation of COVID-19 self-testing among symptomatic presumptive COVID-19 patients?
Future potential of roll-out of COVID-19 self-testing	What do you think would make the COVID-19 self-test acceptable to your community? What do you think are barriers for your community to use the COVID-19 self-test?

*Defined as the perception of self-testing being useful, agreeable or satisfactory in the local context.

^†^Defined as the extent to which COVID-19 self-testing with Ag-RDTs could be successfully implemented, as evaluated by perceived barriers and facilitators for implementation.

Ag-RDT = antigen-detecting rapid diagnostic test.

**TABLE 2:** Themes and supporting quotes

Theme	Quotes
Overarching facilitators	National level Self-testing in an early diagnosis is a step ahead in making the patient well and preventing disease spread or community prevention. Because once you already know that, for example, if you test positive, you take care of yourself. You know who to inform. If not, you will do the measure yourself because you are afraid of infecting your family. And you have to isolate yourself, so it will not spread. So that is a good advantage of self-testing. It’s for early community prevention of disease. [HSDM-002] The advantage is its ability to recommend quarantine to those who tested positive, unlike with RT-PCR, it takes days. Sometimes when the patients still do not know their results, they have tendencies to still go to public places, since they do not know their results yet they do not take extra precautions, but since with antigen test, you can easily get the result and you can recommend or inform the patient to isolate immediately. They can also immediately inform other people thay have been exposed to take the same measures, like testing and isolation, so it would be easier to test, detect an isolation strategy of DOH because of it. [HDSM-009] Provincial level You can have an antigen self-test in the comfort of your home. It will help prevent the transmission of the virus. [HCW-001] Barangay level Because of these test kits, if you have doubts and if you think you have your symptoms, you can test at home first. [HCW-003]
Regulation and reporting	Provincial level The problem is how to regulate it properly and how one can exercise responsibility if you conduct it, particularly having it recorded. You shouldn’t just simply give people 5 of it and then it will not be used. The moment that you use it, it should be reported already and it should be in the repository in the locality or nationwide. Whatever brand it is, it should be reported. [HSDM-003] For example, [if] they are working and they need to quarantine and their company will be asking for a test result, an official result, maybe it will be a hindrance. There might be problems with their return to work since self-antigen tests do now have official results. [HCW-002] Barangay level Of course, they also need a written document that they are negative. How can we give that? Because they won’t do the test if we don’t give them anything. Maybe, official guidelines on how to do it. If I test positive, can I request an official result? That’s what they want. Because their company needs it or in their work. [HCW-004]
Fake tests	Provincial level And I hope that the antigen testing kits also have a regulation because it might be counterfeited. I hope it will be regulated because we are weak in that aspect. There are a lot of fake ones and we do not know how it is being regulated, even with other commodities. So the Filipinos are really good with faking it. [HSDM-008] Int: I will repeat, for it to be acceptable, it should come from a legit source. Res: How would the community know if it is from a legit source? Int: In our health facility. Either here in the city health office or if they would go to their barangay health center, it would be legit since the barangay health centers coordinate with us. If it will come from the staff of the city health office. [HCW-002]
Cost	National level As part of the testing strategy of community or the country since it is very flexible, you don’t need to go to a specific laboratory just to undergo the test and it’s fast, you can easily get the results and it is cheap, it is also more accessible to those who are part of the low income strata, there are a lot of advantages we can maximise if we implement self-testing. [HSDM-009] Provincial level Int: How about the price, Ma’am? Do you think it can be a factor in to use of self-testing? Res: It can be. Especially for those who can’t afford antigen self-testing. It can really be a factor. You can buy it weekly or monthly. But if I can’t afford it, I will not really buy it. [HCW-001] Barangay level They will think that it’s expensive. It will be worth a thousand. That’s what will immediately come to their mind. Instead [of buying a test kit], I’ll just buy [what I need]. That’s what they have in mind. Our barangay is the number one indigent community. [HCW-004]
Regulation	National level You identify what works in their place and it would be difficult if all the penalties and policies in all barangays will be the same. If you look at the demographics of the Philippines, we are different, we have different dialects and we accept these kinds of things differently.[…] So if we really want to push for monitoring, since the self-test kit that we use already provides the immediate results, it should be implemented by the barangay. [HSDM-001] Provincial level Speaking of policy, it would be good if we adapt it in the LGUs because by our experience, if you adapt policy, you really need to tailor fit it with the needs of the LGU. That’s why I think it would be better if we create our own ordinance regarding it, then that ordinance would address and create guidelines on self-testing that works for the LGU emphasising the need to know the legitimacy of the self-test. [HSDM-007]
Linkages to care/digital tools	National level Maybe one of our important learning during COVID-19 pandemic is the use of the digital tools like mobile applications even the internet in general, like laptops but mainly cellphones are being used in rural areas, which can be used for telemedicine which we are actually now doing. Especially, when a patient tested positive doctors or health workers can be contacted through a number and then they can educate the patient on what to do base on the test results so the internet is very critical and also the technological advancement as we move forward with COVID-19 response. [HSDM-009] Provincial level I tried to adapt this one technology I saw from one private medical doctor where everything was digitalised, especially in pulling our medical records, which I think is quite a challenge for us, since we lack staff and of course gadgets and laptops, it is usually sharing with us. So, I think it should be standardised that way even with government hospitals, because honestly, it would really be nice if everything would be ready and efficient for both the patient and the health care worker. [HSDM-007]
Health education	Provincial level Int: Okay, Ma’am. Why did you think the instruction was easy? Res: Because it’s Tagalog. It has Tagalog and English versions. It’s also a step-by-step instruction. It’s clear, and it provides what will I do. If how many drops? […] I think it’s complete. Just instruct the patients to read the instruction carefully because I don’t really read them. Int: Because you already know about it? Res: I really based on what I remember during the demo. But read the instruction carefully. [HCW-001] Barangay level Maybe information dissemination. It must be clear that they will understand. Actually, most people here don’t anymore believe in COVID-19. Once [they have it or] when they have it, or someone dies, they will believe it, and in their mind, it turns out that COVID-19 is real. We already explain it like this and that. It really depends on the explanation. It needs to be friendly and clear. [HCW-004]

RT-PCR = reverse transcription polymerase chain reaction; DOH = Department of Health; HCW = healthcare worker; HSDM = health system decision-maker; LGU = local ­government unit.

Most interviewees perceived self-testing as an acceptable strategy as it is fast, easy-to-use, painless and accessible. Self-testing could facilitate early identification and management of patients, and containment of transmission through routine screening of healthcare workers, labourers in high-exposed or companies in areas where reverse transcription polymerase chain reaction (RT-PCR) testing is not available and newly admitted patients to hospitals. Promoting the protection of family members and friends through self-testing was perceived as a strategy to increase acceptability of self-testing in the general population. Furthermore, interviewees expressed that self-testing could relieve the financial and human resources of current health institutions.

Interviewees indicated that acceptability of self-testing in the general public could be hampered by the perception that COVID no longer exists, or the fear of testing positive because of the subsequent need for quarantine and loss of income. At the time of interviews, some companies mandated the presentation of a COVID-19-negative certificate as a prerequisite for employees to resume work. This certificate, at the time of interview, was only available for facility-based tests. Furthermore, acceptability among interviewees was impeded because of concerns regarding the reliability of self-tests due to the potential use of non-registered tests and perceived erroneous collection of samples that may lead to false-negative results. This was a particular concern for people with lower levels of health literacy.

Interviewees expressed six conditions for implementation. First, self-tests brought to the market should be registered and regulated. Efforts should also be made to remove fake tests from the market. Distribution of legitimate tests could take place through the local community health centres.

Second, interviewees at all levels indicated that for self-tests to be acceptable, they must be cheap or provided free of charge in highly economically deprived areas. Self-testing policy should follow this same rationale as facility-based Ag-RDTs, which are offered free of charge.

Third, more extensive and collaborative policies are needed to guide the implementation and use of self-testing. Some health officers and lower-level decision-makers suggested that new policies by the Department of Health (DOH) should be made in consultation with the stakeholders and community representatives, ensuring that these are acceptable and can be easily adjusted, tailored and enforced at the local government unit (LGU) and community levels.

Fourth, interviewees at the regional level said that the feasibility of COVID-19 self-testing at a programmatic level would depend on the creation of an infrastructure that will facilitate the reporting of COVID-19 self-test-positive persons to the Barangay Health Emergency Response Team (BHERTS). The current reporting system is only designed for the reporting of facility-based Ag-RDT to the national office or the DOH. Several interviewees emphasised the fact that the implementation of self-testing should rely on the consistent allocation of resources to the LGUs and barangays.

Fifth, interviewees said that both feasibility and acceptability of implementing the self-tests would depend on the existence of linkages to care, including a digital referral system. Some expressed the need for confirmatory testing, others said self-quarantine and monitoring of the patient would suffice. Almost all interviewees expressed the need for mandatory reporting and subsequent teleconsultation after self-testing to counsel the individual on the next steps after a positive self-test and to further manage their client. One method suggested was to link QR codes to the self-test kit that could function as a tracking system for the health authorities.

Finally, cultural and linguistically sensitive health education was perceived as crucial for acceptability in terms of uptake of the test by the public, and feasibility in terms of accurate execution of the self-test by the public. Most interviewees perceived community-based, in-person education to be the most feasible and effective. Others suggested the use of social media and/or the barangay radio.

Considering the above conditions for implementation, stakeholders perceived that COVID-19 self-testing was an acceptable screening tool that supports early detection and management of COVID-19. Self-testing is perceived as feasible so long as an effective system that facilitates reporting of test results and provides appropriate linkage to care is developed. This includes guaranteeing the affordability or accessibility of self-tests, conducting informative public awareness campaigns during implementation, and utilising test results to issue certificates for returning to work or gaining admission to hospitals.^5^–^7^ Measures should be taken to address concerns about reliability, such as combating counterfeit tests in the market and incorporating digital tools to enhance testing.^8^,^9^
